# Effectiveness of Ankaferd BloodStopper in Prophylaxis and Treatment of Oral Mucositis in Childhood Cancers Evaluated with Plasma Citrulline Levels

**DOI:** 10.4274/tjh.2017.0320

**Published:** 2018-03-06

**Authors:** Türkan Patıroğlu, Nagihan Erdoğ Şahin, Ekrem Ünal, Mustafa Kendirci, Musa Karakükcü, Mehmet Akif Özdemir

**Affiliations:** 1 Erciyes University Faculty of Medicine, Department of Pediatrics, Division of Pediatric Hematology and Oncology, Kayseri, Turkey; 2 Erciyes University Faculty of Medicine, Department of Pediatrics, Kayseri, Turkey; 3 Erciyes University Faculty of Medicine, Department of Pediatrics, Division of Metabolism, Kayseri, Turkey

**Keywords:** Childhood cancers, Oral mucositis, Ankaferd BloodStopper, Citrulline

## To the Editor,

Oral mucositis is one of the toxic effects of chemotherapy [1]. Ankaferd BloodStopper (ABS) is an herbal product that is used as a hemostatic agent in traditional Turkish medicine. ABS affects the endothelium, blood cells, angiogenesis, cellular reproduction, and vascular dynamics and stimulates the mediators that lead to rapidly progressive wound healing [2]. Additionally, antiinflammatory, antimicrobial, antifungal, and antioxidative effects have been attributed to ABS in previous studies [3,4,5].

In this study, we aimed to investigate the effectiveness of ABS in the prophylaxis and treatment of oral mucositis in patients receiving chemotherapy in childhood. In addition, plasma levels of citrulline, which are a biochemical marker for mucosal barrier injury, were measured and the effectiveness of ABS therapy in mucositis was correlated by quantitative data in addition to clinical assessment.

**Figure 1:**
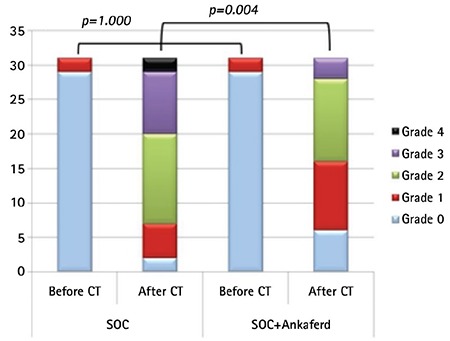
Change in oral mucositis grade before and after chemotherapy treatment between groups. A value of p<0.05 was considered statistically significant. *SOC: Standard oral care, CT: chemotherapy.*

This is a case-control study. The study included 31 patients aged 4-17 years receiving chemotherapy regimens with strong mucotoxic effects. The standard oral care (SOC) protocol consisted of tooth brushing and use of 5% sodium bicarbonate, 0.2% chlorhexidine mouthwash, and nystatin. The patients were asked to perform SOC starting on the first day of a course of chemotherapy, lasting for 14 days, and oral mucosa was assessed daily upon completion of chemotherapy based on the World Health Organization scale for oral mucositis. In addition, blood samples were drawn to measure citrulline levels immediately before initiation of chemotherapy and in the period in which mucositis became most severe. The same patients receiving the same chemotherapeutic agents in the second course of chemotherapy were asked to gargle with ABS (3-4 mL, liquid form) four times daily in addition to SOC. Mucosa ratings were performed before the second chemotherapy course and in the period in which mucositis became most severe. Of the patients included, 17 (55%) were male and 14 (45%) were female. The mean age was 9.3±4.5 years (range: 4-17 years). When the stages of oral mucositis before and after chemotherapy were assessed, it was found that there was no significant difference between chemotherapy sessions given with SOC and with ABS plus SOC before chemotherapy, while there was a significant difference between these sessions after chemotherapy regarding stages of oral mucositis (p=0.004) (Figure 1). When the extent of the decrease in plasma citrulline levels was compared, it was higher in chemotherapy sessions with SOC than in those with SOC plus ABS, indicating a significant difference (p<0.008).

In conclusion, our study is a prospective, clinical trial demonstrating that ABS is effective in the prophylaxis and treatment of oral mucositis secondary to chemotherapy in childhood cancers. Moreover, adding ABS to SOC limits the decrease in plasma citrulline levels. Further randomized studies with larger samples will allow the introduction of ABS in the prophylaxis and treatment protocols of oral mucositis.
